# Research Progress of Carrier-Free Antitumor Nanoparticles Based on Phytochemicals

**DOI:** 10.3389/fbioe.2021.799806

**Published:** 2021-12-08

**Authors:** Siliang Jiang, Yu Fu, Xinyang Zhang, Tong Yu, Bowen Lu, Juan Du

**Affiliations:** ^1^ Department of Pharmacognosy, College of Pharmacy, Jiamusi University, Jiamusi, China; ^2^ School of Clinical Medicine, Jiamusi University, Jiamusi, China

**Keywords:** cancer, carrier-free, nanodrug, phytochemicals, self-assembly

## Abstract

Cancer is a major worldwide public health issue, responsible for millions of deaths every year. Cancer cases and deaths are expected to increase rapidly with population growth, age, and lifestyle behaviors that increase cancer risk. Long-term chemotherapy results in acquired drug resistance. Traditional treatment methods have limitations and cannot effectively treat distal metastatic cancers. Application of nanocarriers in multi-chemotherapy must be promoted. With research progress, the shortcomings of traditional nanocarriers have gradually become evident. Carrier-free nanodrugs with desirable bioactivity have attracted considerable attention. In this review, we provide an overview of recent reports on several carrier-free nanodrug delivery systems based on phytochemicals. This review focuses on the advantages of carrier-free nanodrugs, and provides new insights for establishment of ideal cancer treatment nanosystems.

## Introduction

Cancer is a major worldwide public health issue, causing millions of deaths every year (Siegel et al., 2021). Cancer cases and deaths are expected to increase rapidly with population growth, age growth, and lifestyle behaviors that increase cancer risk (Torre et al., 2016). It is estimated that by 2040, the global cancer burden will nearly double (29–37 million), which will have a significant impact on lower middle-income countries (More et al., 2021). Scientists and doctors have made great research efforts to develop efficient and powerful cancer therapies, including surgery, drug therapy, and radiotherapy (Hashizume and Tsugawa, 2004; Pérez-Herrero and Fernández-Medarde, 2015; Allen et al., 2017). These treatments have their own advantages and disadvantages. Surgical treatment is the first choice for local solid tumors, but its limitations with distal metastatic tumors are obvious (Klein, 2020). With radiotherapy, healthy cells become target cells and are destroyed, resulting in drastic changes in the internal cell environment. Thus, antitumor drugs with curative effects on solid tumors and distant metastases are irreplaceable in cancer treatment. Currently available commercial antitumor drugs can be classified as synthetic drugs or natural drugs. Synthetic drugs are usually the only choice for cancer chemotherapy, but there are problems with drug safety, toxicity, and side effects (Chang et al., 2011). According to the mechanism of action, commonly used chemotherapeutic drugs can be divided into several categories including antimetabolites, alkylating agents, and mitotic spindle inhibitors (Tiwari, 2012; Singh et al., 2018). Gemcitabine (GEM) is a first-line chemotherapeutic drug for pancreatic cancer. However, its curative effect is unsatisfactory. GEM has a short blood circulation time and is dispersed into normal tissues, leading to severe side effects such as nausea and myelosuppression. Moreover, poor uptake, short half-life, and low bioavailability of gem cells require frequent high-dose administration, resulting in severe systemic toxicity (Li et al., 2020c; Takemoto et al., 2020).

Multidrug resistance (MDR) is the main refractory to chemotherapy, defined as the resistance of cancer cells to multiple chemotherapeutic drugs with different structures and mechanisms of action (Li et al., 2017). This obstacle is particularly important because the treatment window for most anticancer drugs is relatively narrow, with a small difference between the dose required to achieve a therapeutic effect and to cause toxicity (Assaraf et al., 2019). MDR can be caused by several mechanisms: decreased uptake of water-soluble drugs; changes in cells that affect the ability of cytotoxic drugs to kill cells, including changes in cell-cycle checkpoints and blocks, an increase in DNA damage repair, a decrease in apoptosis, and changes in drug metabolism; sequestration of anticancer drugs in lysosomes and in intracellular organelles and intercellular vesicles (Szakacs et al., 2006; Li et al., 2016). MDR is responsible for over 90% of deaths in cancer patients receiving traditional chemotherapeutics or novel targeted drugs (Bukowski et al., 2020). According to the biochemical changes in malignant cells, the mechanism of cellular resistance can be divided into non-classical MDR phenotypes and transport-based classical MDR phenotypes (Stavrovskaya, 2000). Non-classical MDR describes non-transport-based mechanisms and includes altered enzyme activity of glutathione S-transferase (GST) and topoisomerase, which can decrease the cytotoxic activity of drugs and changes in the balance of proteins involved in apoptosis (Kartal-Yandim et al., 2016). The classical mechanism targets anticancer drug transport across the cell membrane by increasing the activity of efflux pumps such as adenosine triphosphate (ATP)-binding cassette (ABC) transporters (Liu et al., 2020) to increase the efflux of anticancer drugs through membrane-embedded drug transporters. Membrane-embedded drug transporters are usually overexpressed in cancer cells; recent studies have found that they can be regulated by endogenous cytokines (Kumar and Jaitak, 2019). Although recent studies have explored autophagy to hijack MDR cancer cells in anticancer treatment, the mechanism of the relationship between autophagy and MDR has not been fully studied (Li et al., 2017). Thus, the trend is to use safe natural drugs with tumor growth inhibitory activity that target a variety of cellular pathways in cancer cells with no toxicity to normal cells.

For thousands of years, traditional herbal medicines have provided natural treatment of cancer and many diseases; the discovery of antitumor drugs mainly results from screening of natural products and their analogues (Chang et al., 2011; Salminen et al., 2018). In recent decades, botanical drugs have been widely used in cancer treatment, providing an alternative to traditional treatment with no harmful effects. Although phytochemicals have demonstrated great potential as anticancer agents, many problems remain to be considered. Oral administration of plant preparations may have a first-pass effect and eventually degrade. For example, although curcumin has shown good prospects for cancer treatment, its clinical development is limited due to its low bioavailability and low aqueous solubility. In clinical trials, orally administered curcumin (8 g/day) is rapidly converted into metabolites, resulting in a low level of free curcumin in plasma (<2.5 ng/ml) (Giordano and Tommonaro, 2019). The emergence of drug resistance involving multiple mechanisms is the main obstacle to successful clinical application of phytochemicals as therapeutic cancer drugs. Another obstacle to the use of phytochemicals in clinical practice is the development of MDR (Rizwanullah et al., 2018). The disadvantages of plant compounds such as low aqueous solubility, poor cell permeability, hepatic disposition, narrow therapeutic index, and rapid absorption in normal tissues limit their application (Siddiqui and Mukhtar, 2010). To meet these challenges, the scientific community has turned to the delivery of phytochemicals based on nanodrugs as they can improve water solubility and bioavailability, target specific tumor cell tissues, improve cell uptake, reduce phytochemical dosage, and achieve a stable therapeutic phytochemical level (Dombe and Shirote, 2021; Kim et al., 2021). Although nanotechnology-based drug delivery systems have great potential in cancer treatment, there are still many issues, including low drug encapsulation efficiency and cumulative deposition of nanomaterials *in vivo*. Carrier components usually account for the majority of these nanosystems (Ding et al., 2020). The drug-loading efficiency of anticancer drugs is still very low, and long-term toxicity remains unclear. Use of a large number of carrier drugs to achieve an effective dose leads to low drug-loading and high carrier uptake (Ma et al., 2014; Karaosmanoglu et al., 2021).

Carrier-free nanodrugs used for drug delivery are mainly self-assembled by prodrugs, pure drugs, or amphiphilic drug-drug conjugates. They generally have low systemic toxicity, high drug-loading capability, stimulus-sensitive features, and synergistic therapeutic efficacy (Mei et al., 2022). Recent studies have shown that phytochemicals can form carrier-free nanodrugs through self-assembly. Self-assembly technology can overcome the limitations of phytochemicals in application (Sun et al., 2019; Li et al., 2020a). Phytochemical self-assembled nanodrugs have the following advantages: no carrier; less toxicity and side effects; good drug-loading capacity and pharmacokinetics; MDR inhibition; therapeutic synergy. As an emerging field, research is still in progress. Based on the great progress of carrier-free nanodrugs in cancer treatment, most recent reviews have summarized the preparation, application, and significant antitumor efficiency of carrier-free nanodrugs as a combination of therapeutic agents with different antitumor mechanisms. Carrier-free drugs based on plant compounds have received less attention. In this review, carrier-free drug design based on natural products is summarized to provide a coherent overview and clear direction for the rational design of carrier-free nanodrugs.

## The Preparation of Carrier-free Nanodrugs

Compared with the traditional nanodrug delivery systems, the preparation method of the carrier-free nanodrugs is a simpler process, uses less or no organic solvents are introduced in the preparation process. It is generally divided into *in vitro* and *in vivo* self-assembly strategies. *In vitro* self-assembly strategies include top-down method, reverse solvent precipitation method and template assisted method (Zhang et al., 2015; Gigliobianco et al., 2018). The carrier free nanodrugs prepared by *in vitro* self-assembly strategy can have higher drug loading, longer blood circulation time or more effective absorption. Compared with the *in vitro* self-assembly strategy, the *in vivo* self-assembly strategy utilizes small molecules reaching the target triggered by tumor specific stimulation, which is easier to operate and does not require any high energy consuming machines or other assembly processes (Mei et al., 2022).

Carrier-free drug delivery systems can be rapidly established and used in cancer treatment because of the various attractions of carrier free drug delivery systems; first, Controllable structure, which can protect drugs and improve tumor accumulation; second, increase the drug loading; third, reduce the side effects caused by the carrier; last, simple preparation and administration of drugs (Qin et al., 2017; Shim et al., 2019). The assembly process is a spontaneous behavior of molecules in solvents, which follows the principle of minimum energy. Its essence is intermolecular force. Therefore, compounds that can form non covalent bond force between molecules can usually produce self-assembly. Drug or phytochemical molecules could self-assemble into nanoparticles under the dynamic control of noncovalent bonds, such as hydrogen bond, *π*-*π* interaction, van der Waals force, charge transfer effect, dipole-dipoles and hydrophobic interactions (Whitesides and Grzybowski, 2002; Rybtchinski, 2011; Gao et al., 2016; Yang et al., 2019). When molecules are combined by hydrogen bond, single or multiple hydrogen bond can be formed. Generally, the stronger the multiplicity of hydrogen bond, the stronger the binding energy and stability between molecules. Different organic molecules, such as biocompatible polymeric chains or endogenous molecules, have been used as self-assembly inducers by covalent linkage to drugs. The drug and the self-assembly inducer can be directly attached or can be connected by a linker, which can be stable in serum but able to release the drug intracellularly (Fumagalli et al., 2016).

## Carrier-Free Nanodrugs Based on Curcumin

**FIGURE 1:**
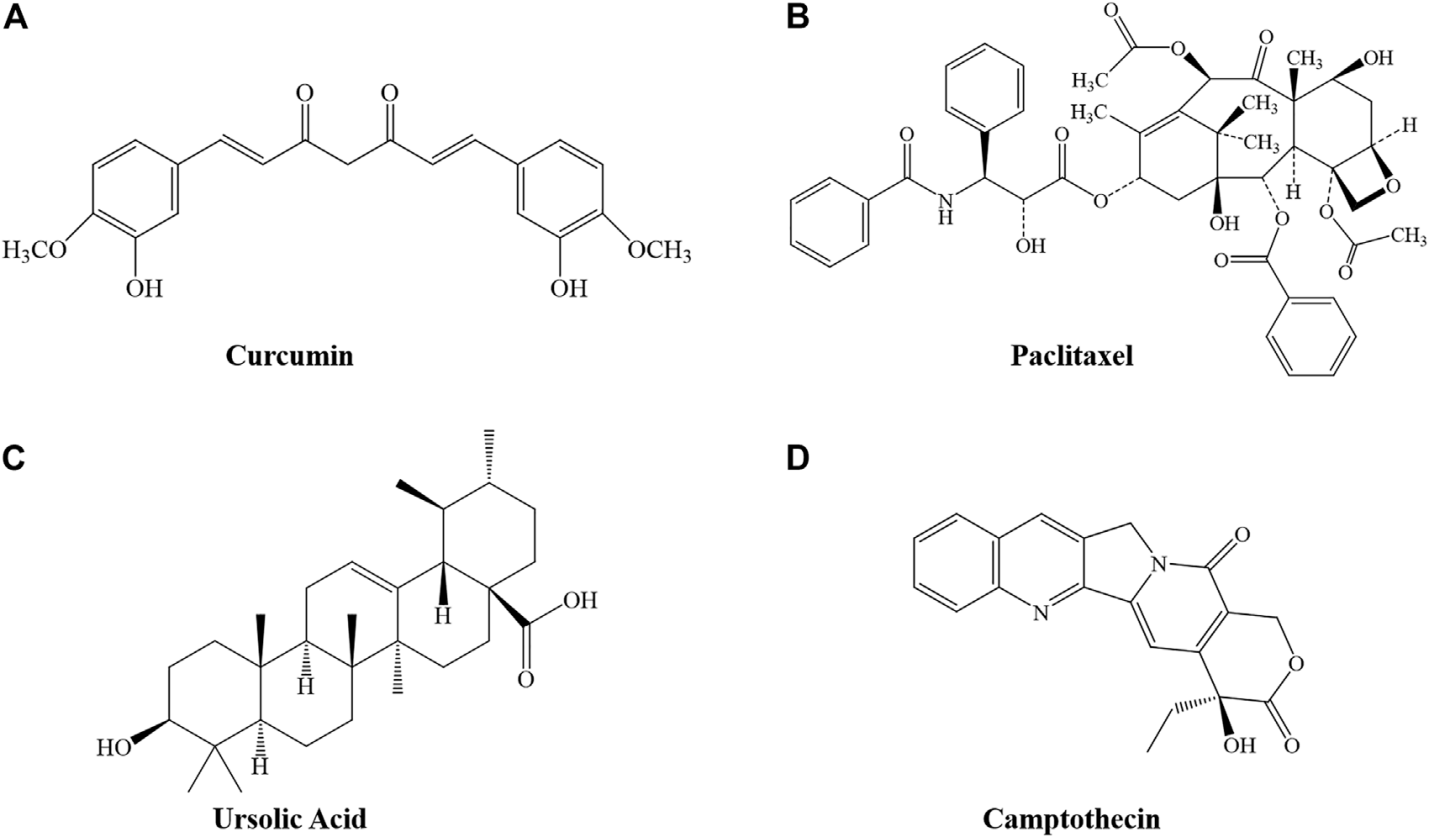
Structure of several phytochemicals. **(A)** Curcumin, **(B)** Paclitaxel, **(C)** Ursolic Acid, and **(D)** Camptothecin.

Curcumin (Cur) (Figure 1A) is a phenolic metabolite isolated from *Curcuma logna* (turmeric), known as diferuloylmethane (Ashrafizadeh et al., 2020; Salehi et al., 2020). The IUPAC name of curcumin is (1E,6E)-1,7-bis(4-hydroxy-3-methoxyphenyl)-1,6-heptadiene-3,5-dione, with a chemical formula of C_21_H_20_O_6_ and a molecular weight of 368.38 (Giordano and Tommonaro, 2019). Cur has demonstrated excellent pharmacological activities, including anti-inflammatory, antioxidant, antitumor, anti-analgesic, neuroprotective, hepatoprotective, and cardioprotective activities (Shabgah et al., 2021). A review of relevant literature confirmed that Cur may have a negative effect on the development, expansion, migration, and invasion of cancer cells (Hu et al., 2018; Pricci et al., 2020; Termini et al., 2020). Similar to other natural plant-derived compounds, curcumin has poor bioavailability, and requires the assistance of a nanodrug delivery system.

Sun et al. (Sun et al., 2019) designed a carrier-free nanodrug (Cur-NDs) based on Cur using the reprecipitation method. The process is based on the strong interaction between Cur molecules (hydrophobic interactions and P-P stacking), allowing formation of stable nanoparticles with sizes of 60–80 nm and retaining the bioactive groups of Cur. Compared with free Cur, Cur-NDs exhibited obvious optical properties and light-sensitive drug-release behavior, resulting in an increase in ROS and photodynamic therapy on breast cancer cells. In addition, apoptosis during Cur-based photodynamic therapy is accompanied by ROS-mediated activation of the JNK/caspase-3 signaling pathway. In addition to self-assembly of Cur molecules, Cheng et al*.* (Cheng et al., 2020) designed carrier-free nanoparticles based on a curcumin-erlotinib conjugate (EPC), with a size of approximately 105 nm and a hydrodynamic size of 146.3 nm with a PDI of 0.157. Compared with free Cur and erlotinib, EPCs have stronger cytotoxicity and better anti-migration and anti-invasion effects on BxPC-3 pancreatic cancer cells. Benefiting from both passive and active tumor-targeting effects, EPCs effectively reduced the growth of pancreatic tumors and extended the median survival time of tumor-bearing mice from 22 to 68 days. Camptothecin (CPT) is an alkaloid with potent antitumor activity first found in plants. CPT analogs (irinotecan and topotecan) have been approved by the FDA for cancer treatment. A recent study designed a self-assembled ion pair complex, ICN, based on Cur and irinotecan (Guo et al., 2021). This unique nanoparticle overcomes the hydrophobicity of Cur, and enhances the antitumor efficacy of irinotecan by integrating a variety of treatments. Liu et al. (Liu et al., 2021) prepared an irinotecan hydrochloride-Cur nanosystem (SICN) in the early stage by a simple precipitation method. SICN NPs had good water solubility and were easily soluble in water and acidic water. The particle sizes of SICN in water and acid water were 61.5 ± 0.22 nm and 50 ± 0.24 nm, respectively. This particle size has little difference in different environments, it could be inferred that SICN are not vulnerable to acidic environment in solid tumors. The results of flow cytometry and zebrafish fluorescence imaging showed that the uptake of SICN was significantly higher than that of free Cur, and the excretion rate was lower. *In vitro* cell experiments showed that SICN NPs were more toxic than single components, while HGC-27 cells had more absorption and higher toxicity to NPs under slightly acidic conditions.

## Carrier-Free Nanodrugs Based on Paclitaxel

Paclitaxel (Ptx, trade name Taxol) is a well-known anticancer agent with a unique mechanism of action (Figure 1B). It is considered to be one of the most successful natural anticancer drugs (Zhu and Chen, 2019). It was approved by the US Food and Drug Administration (FDA) in 1992 for treatment of advanced ovarian cancer (Swain et al., 1995). However, as an aromatic anticancer drug that is usually insoluble in physiological solutions, severely hindering bioavailability, it requires the assistance of a drug-delivery system (Yang et al., 2021).

A recent study reported co-loaded self-assembled nanofibers (P/T-NFs) based on Ptx and tetrandrine (Tet) (Li et al., 2020b). Ptx-SA-RGD obtained by combining Ptx and the tumor-specific peptide arginine-glycine aspartate peptide (RGD) with succinic acid (SA) as a linker was used as a highly effective drug-loaded nanofiber, and encapsulated in P-NFs. Novel P/T-NFs were obtained, mainly formulated through *π*-*π* stacking and hydrophobic interactions. *In vitro* studies have shown that the increased cytotoxicity of P/T-NFs in cancer cells is attributed to an increased level of intracellular ROS, lower expression of p-JAK2 and p-STAT3, and promotion of mitochondrial apoptosis. *In vivo* studies have also confirmed the superior antitumor effect of P/T-NFs, but the mechanism must be further explored. Wang et al. (Wang et al., 2020a) used the antitumor activity and self-assembly characteristics of Ptx and ursolic acid (UA) in nano-preparations (UA-Ptx) by self-assembly. UA-Ptx can effectively prolong the plasma half-life of paclitaxel and ursolic acid and prevent the rapid leakage of drugs in the body. UA-Ptx nanoparticles exhibit the excellent anticancer activity of Ptx, and retain the antitumor and liver-protective effects of UA, with a good synergistic effect. In addition, a novel carrier-free drug delivery system (DDS) assembled from a drug chemical gene conjugate was designed using fluorescent dithiomaleamide (DTM) as a linker to combine two Ptx molecules with floxuridine (FdU)-integrated antisense oligonucleotides (known as a chemogene) (Zhu et al., 2020). This Ptx chemical gene conjugate can be self-assembled into spherical nucleic acid (SNA)-like micellar nanoparticles as carrier-free DDS. It can effectively inhibit the expression of P-glycoprotein, and then release FDU and Ptx to exert a synergistic antitumor effect. Ptx chemical gene conjugates may help reverse MDR in cancer.

## Carrier-Free Nanodrugs Based on Ursolic Acid

UA, or 3-*β*-hydroxy-urs-12-en-28-oic acid (Figure 1C) is a pentacyclic triterpene acid that is abundant in most plants (Yin et al., 2018). Many studies have reported the potential of UA in cancer prevention and treatment; it has great impact in remodeling the tumor microenvironment and can play a direct role in cell proliferation, apoptosis, and cell cycle regulation (Chan et al., 2019; Guo et al., 2019; Khwaza et al., 2020). However, studies have reported that the average plasma concentration of UA is very low, even at high doses, indicating that the bioavailability of UA is low (Liao et al., 2005; Yin et al., 2018).

Carrier-free nanoparticles based on UA-Methotrexate (MTX) have been designed to target folate receptors; their synergistic anticancer activity has been studied *in vitro* and *in vivo* (Lan et al., 2021). The best preparation method for UA-MTX NPs is shown in Table 1. Compared with free UA or MTX, the water solubility of UA-MTX NPs was significantly improved. At pH = 5, the drug release rate of UA-MTX NPs was increased, suggesting that UA-MTX NPs can quickly release MTX in acidic tumor microenvironment conditions. In MCF-7 cells, UA-MTX NPs exhibit excellent folate receptor targeting. In MCF-7 cells overexpressing folate receptors, the antiproliferative capacity of UA-MTX NPs was greater than that of free drugs. *In vivo*, UA-MTX NPs exhibit good biosafety and can improve antitumor efficacy through combined therapy. Song et al. (Song et al., 2014) formulated UA nanocrystals using the antisolvent precipitation method. The results showed that the UA nanocrystals had good aqueous dispensability and could be completely dissolved in 0.5% sodium dodecyl sulfate solution within 120 min. Moreover, the nanocrystal characteristics remained unchanged for 49 days, indicating great stability. Pi et al. (Pi et al., 2016) evaluated the bioavailability of UA nanocrystals and found that it was 2.56 times greater than that of free UA (Shao et al., 2020). Fan et al. (Fan et al., 2018) designed a carrier-free nanodrug through self-assembly of UA molecules. The process was based on the hydrogen bond and hydrophobic interactions between UA molecules. The UA NPs were nearly spherical, with a diameter of approximately 150 nm, and a drug-loading of up to 60%. UA NPs exhibited greater antiproliferative activity, significantly induced apoptosis, decreased expression of COX-2/VEGFR2/VEGFA, increased the immunostimulatory activity of TNF-*α*, IL-6, and IFN-*β*, and decreased the activity of STAT-3 in A549 cells *in vitro*.

**TABLE 1:** Optimization of UA NPs and MTX-UA NPs (Lan et al., 2021).

	Solvent	Concentration of UA (mM)	Methanol/water ratio	MTX/UA ratio
Optimization	Methanol	5	1:5	1:4
Size (nm)	179.3	167.5	143.2	152.6
PDI	0.198	0.114	0.115	0.164
Zeta potential (mV)	—	—	—	−48.2

## Carrier-Free Nanodrugs Based on Camptothecin

CPT (Figure 1D) is a DNA topoisomerase I inhibitor from the Chinese *Camptotheca Acuminat* tree, with excellent anticancer activity in solid tumors including primary and metastatic colon carcinoma, small-cell lung carcinoma, ovarian, breast, pancreatic, and stomach cancers. Poisoning of DNA topoisomerase I is the mechanism by which CPT interferes with tumor growth. Although clinical use of CPT has had a significant impact on cancer therapy, *de novo* or acquired clinical resistance to these drugs is common (Beretta et al., 2013). CPT has low bioavailability, poor water solubility, and significant side effects after injection, hindering further clinical application.

Xu et al. (Xu et al., 2018) connected gemcitabine and CPT through redox-sensitive disulfide bonds to prepare a carrier-free, reduction-degradable Janus prodrug CPT-SS-GEM. CPT-SS-GEM is amphiphilic and can be self-assembled into Janus nano-prodrugs without any excipients in water. The rapid drug release of nano-prodrugs is reduction-dependent. More than 90% of natural CPT and GEM were released in a mimicked tumor cell microenvironment (pH 6.5 PBS, containing 2 Mm DTT) within a period of 3 h. Gao et al. (Gao et al., 2020) connected CPT with Ptx through disulfide bonds to prepare a self-assembled prodrug, PTX-S-S-CPT. PTX-S-S-CPT exhibited good monodispersity, with a particle size of approximately 200 nm. Due to the redox response with a disulfide bond, the PTX-S-S-CPT prodrug NPs significantly inhibited cancer cell growth, with no obvious toxicity to healthy cells. Zhao et al. (Zhao et al., 2020) used CPT derivatives, 7-ethyl-10-hydroxycamptothecin (SN38), and chlorin e6 (Ce6) to co-assemble new carrier-free NPs (SN38/Ce6 NPs) using a simple antisolvent precipitation method. SN38/Ce6 NPs exhibited uniform morphology with a particle size of approximately 150 nm and a zeta potential of approximately −30 mV, good stability in aqueous solution and in a lyophilized state, and high cellular uptake efficiency against murine mammary carcinoma (4T1) cell lines. Wang et al. (Wang et al., 2020b) constructed a new self-assembly based on CPT and carbamoylmannose conjugates (CPT-Man). The self-assembly of CPT-Man formed nanotube components in water with dose-dependent and time-dependent cytotoxicity to MCF-7, HeLa, and A549 cells. In MCF-7 cells, CPT-Man demonstrated potent antitumor activity, with an IC50 value of 2.7 μM in 48 h, an increase of 2.2 times compared with the activity of CPT (6.1 μM).

## Conclusion and Future Outlooks

In this review, the latest progress in carrier-free nanodrugs based on several anticancer phytochemicals was summarized. With research advances, the advantages and versatility of carrier-free nanodrug systems for cancer treatment are gradually becoming evident. NPs based on a phytochemical self-assembly strategy use bioactive natural drug components with self-assembly characteristics as the carrier without introducing other materials, improving drug delivery efficiency, exerting drug efficacy, and reducing toxic and side effects. Self-assembled NPs of different phytochemical molecules can play a synergistic role in treatment for a variety of diseases. The properties of hydrophobic drug molecules can be easily adjusted by combining them with functional molecules to prepare nanodrugs with improved functional properties. Different self-assembly behaviors are observed based on the strength and nature of non-covalent interactions such as van der Waals forces, hydrogen bonds, and *π*-*π* stacking, resulting in different nano-assembly sizes and morphologies (Karaosmanoglu et al., 2021). However, there are still some defects in carrier-free NPs based on self-assembly of phytochemicals. Most current NPs are limited to a combination of two drugs; there are few reports on self-assembly of more drug molecules. In addition, the self-assembly force between phytochemicals is based mainly on intermolecular forces; thus, the stability of NPs is poor. Further, NPs should be tuned to optimize their key properties including size, shape, and surface chemistry. Carrier-free drugs have shown promising results in overcoming MDR. However, the potential biological toxicity of carrier-free drugs has not been clinically verified; much research is needed for confirmation. The authors are optimistic that in the near future, carrier-free drugs based on phytochemistry will find a place in treatment of drug-resistant cancer.
